# From Self-Assembly of Colloidal Crystals toward Ordered Porous Layer Interferometry

**DOI:** 10.3390/bios13070730

**Published:** 2023-07-13

**Authors:** Yi-Zhen Wan, Weiping Qian

**Affiliations:** 1 State Key Laboratory of Digital Medical Engineering, School of Biological Science and Medical Engineering, Southeast University, Nanjing 210096, China; 2 OPLI (Suzhou) Biotechnology Co., Ltd., New District, Suzhou 215163, China

**Keywords:** self-assembly, colloidal crystals, reflectometry, optical interferometry, ordered porous layer interferometry, biosensors, label-free

## Abstract

Interferometry-based, reflectometric, label-free biosensors have made significant progress in the analysis of molecular interactions after years of development. The design of interference substrates is a key research topic for these biosensors, and many studies have focused on porous films prepared by top-down methods such as porous silicon and anodic aluminum oxide. Lately, more research has been conducted on ordered porous layer interferometry (OPLI), which uses ordered porous colloidal crystal films as interference substrates. These films are made using self-assembly techniques, which is the bottom-up approach. They also offer several advantages for biosensing applications, such as budget cost, adjustable porosity, and high structural consistency. This review will briefly explain the fundamental components of self-assembled materials and thoroughly discuss various self-assembly techniques in depth. We will also summarize the latest studies that used the OPLI technique for label-free biosensing applications and divide them into several aspects for further discussion. Then, we will comprehensively evaluate the strengths and weaknesses of self-assembly techniques and discuss possible future research directions. Finally, we will outlook the upcoming challenges and opportunities for label-free biosensing using the OPLI technique.

## 1. Introduction

In recent years, label-free biosensing techniques have garnered significant interest due to their simple structures and ability to provide real-time, in-situ detection [1]. Through monitoring biomolecular interactions in real-time and in-situ, more comprehensive kinetic information, including affinity, can be obtained compared to the molecular concentration information that has been the primary focus of most biosensors. In such sensors, the reflectometric biosensors based on optical interferometry are a type of label-free biosensor with great potential for development. Although their application scenarios were limited in the early stages, they have been continuously developed and broadened for many applications, especially in the fields of life science, public health, and biomedicine, and have achieved success in commercial detection (for example, biolayer interferometry, or BLI) [2].

For reflectometric interference sensors, there are two types of interference substrates: planar and porous substrates. Porous substrates have been extensively studied due to their larger internal surface area compared to planar substrates, which provide more molecular binding sites and thus yield a stronger signal response. In early studies, most of the porous interference substrates were obtained using top-down methods, such as electrochemical etching techniques, which are represented by porous silicon (pSi) and anodic aluminum oxide (AAO) [3,4]. These two substrates have not only high porosity but also a high refractive index, which can produce a strong interference signal. However, due to the randomness of electrochemical etching, the repeatability of the pores is relatively poor. Moreover, the pSi and AAO exhibit low transparency to white light, resulting in incident and reflected lights located on the side of the test sample. This can significantly affect accuracy due to variations in the color or turbidity of fluidic samples.

In recent years, ordered porous interference substrates fabricated by self-assembly techniques (bottom-up methods) have been utilized in interferometry biosensing applications, providing a novel avenue for the study of porous interference substrates [5,6]. Self-assembly has been successfully achieved in laboratory settings by recreating the fascinating thermodynamic mechanisms found in nature. This fundamental biological design process enables the formation of living organisms from smaller components without external guidance. The scientific community is continuously deepening its understanding of self-assembly systems, striving to translate physical mechanisms into innovative materials synthesis. Scientists have gained sufficient expertise in exploiting this phenomenon to design and fabricate entirely novel nanomaterials and microsystems.

Self-assembled nanomaterials have become more attractive in recent years [7]. The reason for this could be summarized in two points: One is the fact that traditional top-down fabrication methods for nanomaterials require very expensive equipment like lithography machines or ion etching equipment, and the whole process is quite complicated. As a bottom-up fabrication method, self-assembly can offer a budget-friendly cost and simplicity for nanomaterial synthesis through simple components and procedures [8]. Another advantage is that self-assembly can create materials and devices with unique behaviors and properties that are difficult to achieve by other methods. Colloidal crystals (CCs) are a type of optical nanomaterial with a periodic, ordered structure that can provide unique optical properties. CCs can be constructed through various self-assembly methods, such as vertical deposition [9], Langmuir-Blodgett (LB) method [10], spin-coating [11], external-force-driven assembly [12,13], and microfluidic methods [14,15].

**Figure 1:**
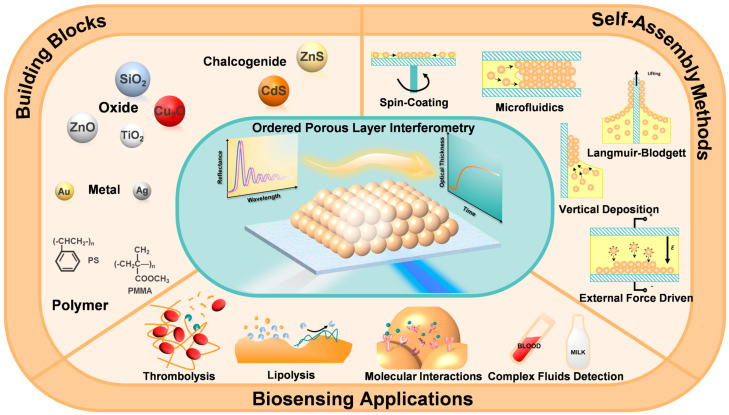
Schematic diagram illustrating the contents of this review, including building blocks for self-assembly, self-assembly methods, and biosensing applications of OPLI.

In recent years, many reviews related to self-assembly have summarized the types of materials, fabrication methods, and traditional optical applications [8,16]. There are also interferometric sensor-related reviews summarizing and comparing the applications of different classes of interferometric sensors [1,2]. This is the first comprehensive review of the use of ordered porous CCs in the field of interferometry. From the perspective of self-assembled materials, this review will introduce various building blocks used for CCs’ self-assembly, summarize their self-assembly methods, and discuss the advantages and disadvantages of each method. The versatile biosensing applications are achievable through the combination of self-assembled colloidal crystals as porous interference substrates with reflectometric interferometry, which is called ordered porous layer interferometry (OPLI) (see Figure 1). Finally, we will provide a comprehensive overview of the benefits and drawbacks associated with utilizing ordered porous films in reflectometric interferometry, as well as outline potential avenues for its future development.

## 2. Building Blocks of Self-Assembled CCs

The synthesis of building blocks, or nanoparticles, is fundamental for self-assembly. There have been various chemical methods that produce nanoparticles in different sizes and shapes [16,17,18]. The most commonly utilized materials for self-assembly are monodisperse nanoparticles, which exhibit a spherical morphology that facilitates close and regular stacking. Additionally, our survey revealed the presence of composite structures such as core-shell nanoparticles, which display distinct properties from homogeneous materials.

In this section, we investigated how to synthesize the most popular building blocks, including oxides, metals, chalcogenides, and polymers.

### 2.1. Oxide Spheres

The most common building blocks for CCs are monodisperse SiO_2_ spheres, and the first well-known method for this was published by Stöber’s group. The Stöber method is a typical sol-gel process wherein a tetraethyl orthosilicate (TEOS) is first reacted with water in an alcohol solution, and the resulting siloxane groups then join together to build larger structures [19]. These particles present a surface charge due to the ions in the synthesis system. With proper control of pH, temperature, and concentration of components, very monodisperse SiO_2_ spheres can be obtained from a few nanometers to a few micrometers [20]. The kinetics and mechanism of the drug have been investigated since its discovery. The particle aggregation model proposed by Bogush et al. was found to be a better fit for the experimental data [21]. The final size of spheres depends on the balance between the nucleation process and the aggregation process. This further understanding enabled scientists to fine-tune the particle size and distribution. 

**Figure 2:**
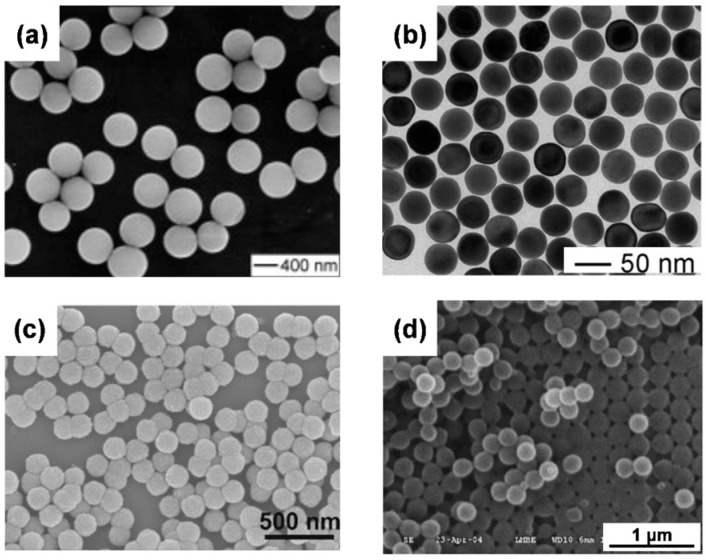
Electron microscopy images of various monodisperse nanospheres. (**a**) Scanning electron microscope (SEM) of TiO_2_; Adapted with permission from [25]. Copyright (2003), John Wiley and Sons. (**b**) Transmission electron microscope (TEM) image of Au. Adapted with permission from [35]. Copyright (2013), John Wiley and Sons. (**c**) SEM image of ZnS. Adapted with permission from [36]. Copyright (2022), John Wiley and Sons. (**d**) SEM image of poly(methyl methacrylate) (PMMA). Adapted with permission from [37]. Copyright (2007) Elsevier B.V.

To obtain spheres with a higher refractive index and different properties, metal oxide monodisperse spheres were frequently studied. The Stöber method has been used to generate metal oxide as well. Titanium dioxide (TiO_2_) is a good material that is used in optoelectronics. Barringer and Bowen synthesized monodisperse TiO_2_ by hydrolysis of titanium alkoxides in an alcohol solution [22]. After this, Jean et al. studied the nucleation and growth mechanisms of TiO_2_ within the alcohol solution and reported the proper nucleation rate and growth rate to generate monodisperse spheres [23]. To avoid the aggregation of TiO_2_ spheres during the generation of titanium alkoxides, Look et al. increased the ion concentration to stabilize the particle [24]. Further, Jiang et al. significantly slowed the hydrolysis rate of the conventional sol-gel precursor by forming a coordination complex with ethylene glycol. And this method could synthesize uniform titanium glycolate CCs (see Figure 2a), which can easily convert the titanium glycolate into anatase or rutile by just annealing these spheres at elevated temperatures in the air [25]. In more recent research, more stable and monodisperse TiO_2_ can be obtained by using thioglycolic acid as ligands to stabilize the titanium alkoxides and increase the charge density on TiO_2_ spheres [26]. 

In addition, Cu_2_O is an ideal candidate for high refractive index (above 2.7) CCs. Zhang et al. reported a method to synthesize single-crystalline Cu_2_O spheres with the reduction of Cu(CH_3_COO)_2_ with NaBH_4_ as the reductant and polyvinyl pyrrolidone as the capping agent at 85 °C–95 °C [27]. Bi and coworkers proposed a room-temperature isotropic growth strategy to prepare monodisperse, single-crystalline Cu_2_O spheres [28].

Another popular photonic material is zinc oxide (ZnO), which shows high-efficient ultraviolet (UV) emission and can be used as a laser device. The ZnO nanospheres can be synthesized by hydrolysis of zinc salt in a polyol medium, as proposed by Jézéquel et al. [29]. The relatively higher boiling points of polyols can provide a wide temperature range for inorganic compounds. Seelig et al. reported a two-step method, modified from the Jézéquel method, that can provide monodisperse ZnO spheres over a broad size range from 100 nm to 600 nm with good control [30].

Despite the materials discussed above, ceria is another unique semiconductor, and its oxides (CeO_2_) are widely used in heterogeneous catalytic reactions [31]. Nguyen et al. reported a two-step hydrothermal method to form CeO_2_ nanospheres [32]. Tuyen et al. modified Nguyen’s method to synthesize monodisperse CeO_2_ nanospheres and evaluated their catalytic performance [33]. Besides, hollow-structured monodisperse CeO_2_ spheres were produced by Yang et al. to gain better catalytic properties at higher temperatures [34].

### 2.2. Metal Spheres

Since metal materials tend to crystallize to grow anisotropically into nonspherical shapes, metal nanospheres larger than 100 nm were hard to synthesize, and large monodisperse colloidal spheres were traditionally limited to amorphous materials such as silica or polymers. Goia et al. summarized the preparation methods of monodispersed metal particles from nanometer to micrometer size and especially emphasized chemical precipitation from solutions [38]. Yakutik et al. synthesized monodisperse silver (Ag) nanospheres in the range from 150 nm to 1500 mm using K-Na-tartrate as the reductant for Ag^+^ in the presence of gelatin [39]. Wang and Xia presented a bottom-up (thermally decomposing metal acetate in boiling ethylene glycol) and a top-down method (emulsifying molten drops of metal in boiling di(ethylene glycol) that can synthesize monodisperse metal spheres with melting points below 400 °C, such as Bi, Pb, In, Sn, and Cd [40]. Zheng and coworkers reported a robust method to synthesize single-crystal gold (Au) spheres in the range from 5 nm to 150 nm with Au seeds (refer to Figure 2b) [35]. Those reports laid a solid foundation for high refractive index CCs.

As our investigation goes on, we find out that conventional solid-structure metal spheres with relatively large sizes were reported less recently. To provide novel properties in self-assembly, encapsulation, photoelectronics, and catalysis, porous, core-shell, hollow, and Janus structures were studied more and more. Camargo et al. described a cation exchange approach to the synthesis of core-shell metal spheres that can be used as multifunction materials [41]. Qiu et al. reported a method to synthesize noble metal spheres in Janus structures that shows interesting photonic properties [42]. These structures have been detailed in another specific review [18].

### 2.3. Chalcogenide Spheres

Metal sulfide semiconductors have important photoelectron properties that can be applied to photocatalysts, field effect transistors, and lasers. These monodisperse spherical metal sulfides can also be synthesized by the methods described in the oxide spheres. For example, the hydrothermal method can still be used to prepare zinc sulfide (ZnS) monodisperse spheres. Kim et al. proposed the method by which zinc nitrate heated with thioacetamide (a sulfur source) can synthesize ZnS monodisperse spheres ranging from 100 nm to 900 nm and reported the sintering behavior of ZnS nanospheres [43]. Wang et al. prepared ZnS nanospheres by heating zinc acetate dihydrate with thiourea at 140 °C for 8 h [44]. Fang et al. reported a two-step method to precisely control the size of ZnS spheres; this method includes the hot injection process to obtain seeds and the heating-up process for seed-mediated aggregation growth (see Figure 2c) [36].

Another important chalcogenide nanomaterial is cadmium sulfide (CdS), which has been widely used in solar cells and lasers. Rodríguez-Castañeda et al. synthesized monodisperse CdS spheres with a hydrothermal method, slightly modified by using a microwave oven to heat the reaction tube [45]. CdS prepared by the solvothermal procedure can provide highly monodisperse spheres with precise tunable sizes from 80 nm to 500 nm [46]. This method allows highly concentrated monodisperse CdS nanospheres to be synthesized, which meets the requirements of practical applications. Xu and Han reported a mild liquid-liquid interfacial synthesis method that can prepare CdS monodisperse spheres at the interface between CS_2_ and dimethyl sulfoxide at 80 °C, but this method takes a much longer reaction time than the traditional synthesis method [47].

### 2.4. Polymer Spheres

Polymeric spheres are widely used in CCs, and polystyrene (PS) and PMMA are the two main materials. The synthesis techniques for these two nanospheres are quite a lot, such as precipitation polymerization, emulsion polymerization, emulsification–solvent evaporation, emulsification–solvent diffusion, and emulsification–reverse salting-out [48]. Those methods can produce polymeric spheres either from in-situ polymerization or preformed polymers. 

Based on those basic methods, many improvements have also been proposed in recent years. Gu et al. prepared a series of polymer monodisperse spheres by boiling-temperature soap-free emulsion polymerization that makes the preparation process easy to apply in the laboratory and industry [37]. Liu et al. extended the classical Stöber method that is most commonly used in preparing inorganic nanospheres to synthesize the resorcinol formaldehyde (RF) resin monodisperse spheres [49]. Al Najjar et al. refined the reaction parameters of emulsion polymerization and obtained a high concentration of PS monodisperse spheres without any filtration or centrifuging [50]. Wang and coworkers reported a solvent/antisolvent self-assembly method that narrowed down the dispersion of lignin spheres [51].

In recent years, as the requirements for non-toxic materials in biochemistry or bio-diagnostics and the performance of electronics have improved, the study of carbon monodisperse spheres has also improved. An efficient synthesis route involves transforming polymer spheres into carbon spheres. Zhao et al. synthesized carbon spheres by the autocatalysis of poly(benzoxazine-co-resol) monodisperse spheres [52]. Ghimire et al. prepared polyvinyl pyrrolidone-stabilized RF polymer monodisperse spheres by a Stöber-like method and carbonized them to obtain carbon spheres [53]. Yu et al. synthesized internal-gridded hollow carbon spheres under a controlled pyrolysis microenvironment from RF polymer spheres [54].

## 3. Self-Assembly of CCs

**Figure 3:**
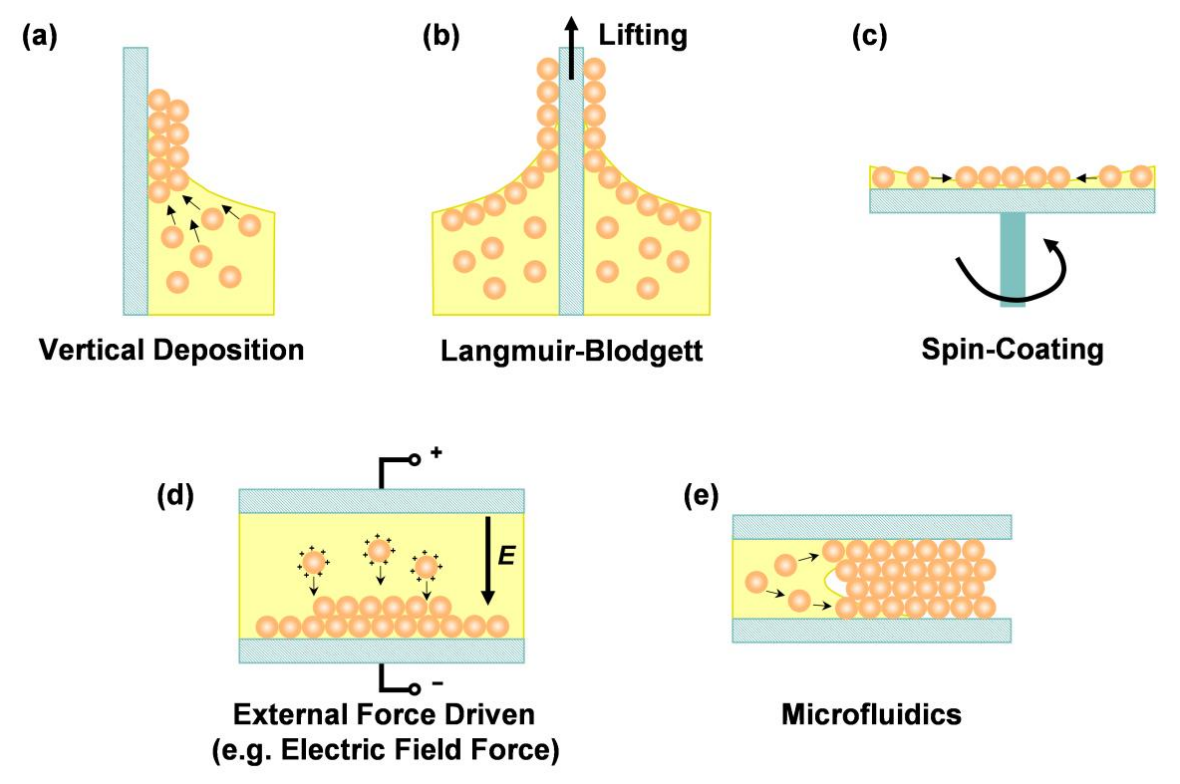
Schematic diagram of self-assembly methods for CCs. (**a**) Vertical Deposition. Nanoparticles are self-assembled by the capillary force on the meniscus. (**b**) LB method. Surface tension is controlled to form a colloid monolayer on the solvent interface, and the monolayer can be transferred to the substrate by lifting it. (**c**) Spin-coating method. Nanoparticles self-assemble with centrifugal force and solvent evaporation. (**d**) Self-assembly by external force. (**e**) Microfluidic method. Nanoparticles self-assemble in the microchannels.

The fabrication of large-area CCs with high quality is the key challenge for real-world applications. There are two main routes to fabricating CCs, namely top-down and bottom-up methods [8]. The micromachining technique is representative of traditional top-down methods, which provide precise control of structure and defects. While providing such advantages, this route also has high costs and complex processing disadvantages. The bottom-up method is based on the self-assembly of building blocks, providing a much simpler and more budget-friendly way to fabricate 2D or 3D CCs. Various self-assembly methods have been developed to create CCs with different structures, symmetries, and functionalities. We summarized the self-assembly approaches to fabricating CCs into five sections: vertical deposition, LB method, spin-coating, self-assembly driven by external forces, and microfluidic methods. (Figure 3).

The quality of CCs is people’s main focus, so defects such as cracks, vacancies, and boundaries always attract attention during large-area self-assembly fabrication [55]. The most common defect is cracking in CCs after the film fabrication, which is mainly due to the capillary stresses in the preparation and further cracks brought on by drying or sintering treatment [56]. Those cracks further prevent the application of self-assembled CCs in the field of high-quality optical devices. As our investigation goes on, most of the articles that describe an improved self-assembly method are trying to solve the large-area defects and shorten the fabrication time of CCs. In the following sections, we will introduce those improvements developed for high-quality self-assembly CCs in recent years as well.

### 3.1. Vertical Deposition

The vertical deposition method is a conventional approach for high-quality and mechanically stable large-area CCs films, but the disadvantage is that the preparation speed is slow. The evaporation rate and particle sedimentation rate are essential to vertical deposition. It is still a classic method to prepare high-quality CCs at present. This method relies on capillary forces to organize building blocks to fabricate 3D CCs. At the interface of substrate, air, and solvent, the meniscus allows nanospheres to self-assemble through capillary forces, and the film starts to grow as the liquid level goes down because of solvent evaporation. (Figure 3a) Colvin’s group first reported this method systematically for controlling the number of colloidal layers and the effect of sample thickness on the optical spectrum [9]. By this method, the close-packed ordered structure CCs can be prepared, and the film can be deposited several times at the same position to increase the thickness successively up to 50 μm. This work demonstrated that film thicknesses ranging from single monolayers to 100 layers are easily created through control over the particle volume fraction and size of spheres. 

Despite those pathbreaking methods, researchers are making efforts to improve them. As we mentioned before, cracks are the biggest restriction to the application of CCs in high-performance optical devices. Wang and Zhao reported a template-free method for the fabrication of large-area crack-free CCs by the vertical deposition method [57]. A silica precursor solution (including TEOS) is added to the colloidal solution, and during the self-assembly of silica spheres, additional silica is formed from the hydrolysis of TEOS. Then excess silica will be removed by hydrofluoric acid (HF) vapor and left with crack-free CCs films. Although the addition of TEOS lowers the capillary stresses, leaving a crack-free film during self-assembly, the CCs show non-close-packed morphology after the etching by HF vapor, which is unacceptable for high-quality optical devices. Zhang et al. tried to improve the vertical deposition in another aspect [58]. They use negative pressure to reduce the surface tension of colloidal solutions and increase the wettability of the substrate, which facilitates the fabrication of continuous, uniform CCs films. On the other hand, the negative pressure will accelerate the solvent evaporation rate, resulting in the growth time decreasing greatly and overcoming the rapid gravity-driven sedimentation of large spheres. This method nearly solved the three pain points of vertical deposition with just negative pressure and extended the robustness of vertical deposition under the polydispersity, impurity, and surface roughness of colloidal spheres. However, this method cannot solve the problem of defects in self-assembly.

The concentration of colloidal spheres changes during solvent evaporation, which could affect the thickness uniformity of CC films. Gu et al. changed the strategy of vertical deposition by lifting the substrate from the solution vertically at a constant speed instead of relying on the liquid level dropping from solvent evaporation [59]. This is named the dip-coating method after its publication. Dip-coating solved the problem of concentration change during fabrication and avoided the rapid sedimentation of large-size spheres. By controlling the lifting speed and the volume fraction of the colloidal solution, good repeatability of film thickness can be obtained. The only drawback is that a significantly higher colloid concentration is required to achieve thick films compared to vertical deposition. However, this may facilitate the production of 2D monolayer CCs with greater ease at low colloid concentrations. Subsequently, this method became a popular approach to rapidly fabricating CCs. Kim et al. limited the colloidal self-assembly inside two parallel substrates close to each other by lifting one of the substrates [60]. They used this method to fabricate 2D or 3D PS CCs successfully with a relatively low colloid concentration, and this report also showed its ability to make binary CCs that can be used as advanced masks for photolithography. Colosqui and coworkers delved into the hydrodynamics of the dip-coating, which provides the theoretical guideline for this method [61]. A much faster lifting rate, about 1 mm/min, to fabricate 2D or 3D CCs by surfactant induction was achieved by Armstrong et al., which accelerated the fabrication speed by an order of magnitude [62]. In a recent application, Bai et al. used the dip-coating method to fabricate photonic films with low brightness/angle dependence [63], which shows the vitality of the dip-coating method.

In addition to the traditional flat substrate, vertical deposition can also be applied to the curved substrate. Zhao et al. proposed an interesting bioinspired application that fast-self-assembles colloidal spheres in capillaries [64]. By adjusting the parameters, the width, spacing, and color of the stripe patterns can be precisely controlled to establish structure color codes. Cheddah et al. studied the hydrophobic moieties in the assembly of silica colloidal crystals in capillaries [65].

### 3.2. Langmuir-Blodgett

The LB technique is another alternative to CC self-assembly. It is similar to the conventional LB method, which is to fabricate monolayer molecules at the air/liquid interface and transfer them onto a substrate. The LB technique enables the rapid fabrication of large-area 2D CCs by lifting the substrate from a colloid solution. While similar to dip-coating, there is a critical difference between them: in the LB technique, a colloid monolayer is already formed at the air/liquid interface (as shown in Figure 3b), whereas self-assembly via capillary forces occurs at the substrate/liquid/air interface in dip-coating. By applying the LB technique several times, 3D CCs can also be fabricated. Fulda and Tieke first reported the application of the LB technique in fabricating monodisperse polymer nanospheres into 2D CCs [10]. They characterized the nanosphere monolayer at the air/water interface by measuring the surface pressure-area isotherms and then transferred the monolayer onto a glass or aluminum substrate. This was progress that solved the problems of the long fabrication time and poor thickness repeatability of the traditional sedimentation method at that time. The constant surface pressure assured the concentration of colloids at the air/water interface, which further allowed thickness homogeneity, and the CCs with larger-sized spheres could also be prepared. However, the crystal structure fabricated by the LB technique cannot be concluded to be a well close-packed structure. A lot of vacancies and quadrangular packing structures were shown in the SEM figures. van Duffel et al. dispersed silica spheres in a sodium dodecyl sulfate chloroform, or ethanol solution as the suspension for the LB method, which increased the crystallization of CCs films [66]. The film quality was noticeably better than what Fulda and Tieke fabricated first. They further studied the lattice constant of the 2D opal structure by AFM. However, the film quality was also far worse than what vertical deposition can achieve.

Reculusa and Ravaine successfully synthesized allytrimethoxysilane-functionalized silica spheres that conferred the hydrophilic-hydrophobic balance of the silica spheres, which is critically important to the close-packed hexagonal organization of the silica colloid 2D array at the air/water interface [67]. The investigation of crucial parameters in the LB technique to fabricate high-quality 2D CCs recently also confirmed the importance of surface modification on nanospheres [68]. This work improved the quality of CCs fabricated by the LB technique greatly. The 2D silica CCs can be extended to the 3D structure by applying this method several times up to 25 layers on different substrates (including glass, CaF_2_, and mica). They applied the Bragg diffraction law and scalar wave approximation to fit their experimental data and found high consistency. Although there were some vacancies and cracks on the films, this report still provided a solid foundation for the LB technique to rapidly fabricate CCs to move from the laboratory to practical large-scale applications.

In recent years, some interesting engineering techniques have been proposed to assist the LB method in fabricating large-scale 2D or 3D CCs, and the roll-to-roll method is one of them [69,70,71]. Parchine et al. applied the roll-to-roll technique to the LB method and fabricated 2D silica CCs on flexible poly(ethylene terephthalate) (PET) film with a total area of 340 cm^2^ [69]. The different sizes of silica spheres should be treated differently because larger spheres (over 400 nm) needed surfactant to float at the air/water interface and smaller spheres did not. They also fabricated 3D CCs by using this method multiple times, and the crystal quality was good enough for further application. In another article, Parchine et al. further applied this roll-to-roll LB technique to fabricate flexible organic photovoltaic modules that can increase the photoelectric conversion efficiency of solar cells [70]. This was the first application of large-area 2D CCs for light trapping in commercial solar cell modules by this low-cost fabrication method. By applying this technique, a specific range of the solar spectrum could be enhanced to improve efficiency. In the lithography field, Chen et al. used this roll-to-roll technique to fabricate 2D CCs as the continuous phase element for near-field nanolithography, which developed 3D periodic nanostructures on a photoresist. This can be a simple approach to producing continuous, large-scale, periodic 3D nanostructured masks.

### 3.3. Spin-Coating

As previously discussed, fast, highly uniform, and industrial-scale massive fabrication cannot be achieved by such gravity-sedimentation or vertical deposition. Those fabrication processes do not match the demands of wafer-scale batch pipelines in the semiconductor industry. If the CCs are embedded in the manufacture of modern photoelectric devices, the thickness and crystalline structure should be highly controlled on a large scale. Against this background, a wafer-size 3D CC fabrication method was proposed by Jiang and McFarland [11]. This was the first time that the spin-coating method, which is widely used in thin-film preparation, was introduced in the fabrication of 3D CCs. Previously, spin-coating was applied in a stepwise manner in the fabrication of 2D CCs or binary structures [72,73]. Unlike the capillary force used in vertical deposition, the spin-coating method uses shear-induced crystallization to fabricate CCs. In this report, they controlled the film thickness by tuning the spin-coating speed and time, and the film can be fabricated in several minutes. Despite that, the SiO_2_ spheres were dispersed in ethoxylated trimethylolpropane triacrylate (ETPTA) monomer with a photoinitiator, which can provide mechanical stability by photopolymerizing after the spin-coating. The ETPTA or SiO_2_ would be removed based on the porous structure needed. In particular, after removing the SiO_2_ spheres, the microporous ETPTA polymer could be processed by lithography to microfabricate the micropatterns. Surprisingly, there were no obvious defects like vacancies or cracks under low-magnitude SEM images. However, the structure described in this article was not close-packed. The volume fraction of SiO_2_ spheres in the nanocomposite film is only about 52.3% rather than 74%, which is in good agreement with Bragg’s law. This phenomenon was explained by the vertical compression of the shrinkage of ETPTA after polymerization. Besides, the non-volatile monomer solvent limited the choice of nanoparticles, to which the monodisperse latex nanospheres could not be directly applied because they would be removed simultaneously in the etching step. This method made the integration of 3D CCs into microfabrication possible, which provided a route for large-scale CC fabrication.

The solvent used in Jiang’s report was non-volatile, which might have a critical influence on forming a close-packed structure and further processing. Mihi et al. investigated the CC structure formed by the spin-coating method in a volatile solvent [74]. In this study, ethanol, water, ethylene glycol, and their mixtures were chosen to disperse colloidal particles more easily. By controlling the evaporation rate of solvent, different packing structures can be obtained, which indicates that not only the shear but also the capillary force participated in the self-assembly. Eventually, the close-packed CC structure can be obtained by using specific nanosphere and solvent ratios. Yet, the defects (vacancies and cracks) were shown in the SEM images, which might be due to the fast evaporation. This was huge progress after Jiang’s research because they reduced the selective etching process, achieved a close-packed structure, and introduced more types of building blocks. To reduce those defects, Colson et al. proposed a method that was particularly applicable to spin-coating 2D CCs [75]. They analyzed the experimental parameters that affect crystal quality and found a protocol that could obtain large-crack-free domain (200 μm^2^) CCs.

The dynamic process of CC structure in spin-coating was also deeply studied. Giuliani et al. first reported the dynamic of evaporative colloid spin-coating, which revealed the internal mechanisms of shear-induced fabrication [76]. They combined high-speed imaging, AFM, photography, and SEM to determine the structures. The patterns shown on the wafer were captured by a high-speed camera, and the very transient dynamic was able to be studied. Under different rotation speeds, different zones on the wafer showed either four-arm or six-arm and as the film dried, the transitions of the pattern were observed. This phenomenon has an interpretation in the center-position, non-close-packed crystal structures reported before. They found four different phases in the transient dynamics, which could guide further research on spin-coating-fabricated CCs. As the film thinned, local volume fraction and stress profiles were controlled by the thinning dynamics, driving the structural transitions.

It can be inferred that spin-coating has made the large-scale production of CCs a reality in the industry. Nevertheless, the crystal quality is currently only adequate and not sufficient for the high optical demand of micro-optic devices. Further investigation into improving the quality of spin-coated CCs could be critical to future mass production.

### 3.4. Self-Assembly Driven by External Forces

The initial self-assembled CCs are prepared by gravity-driven sedimentation, which makes it hard to control the crystal structure and thickness. Even in this condition, Mayoral et al. made some efforts to increase the quality of the CCs obtained by natural sedimentation [77]. The sedimentation rate of colloids is significantly affected by the size of nanospheres, and the sedimentation rate is slow when the size is too small or too fast to form an ordered structure when the colloid size is too large. To control the sedimentation rate, other external forces were studied to fabricate ordered CCs, such as electric field force, surface tension, or magnetic field force.

The electric field force was first used to fabricate opal CCs. Holgado et al. introduced the electrophoretic concept to opal colloidal fabrication [12]. They set up a device that allows direct current voltage to control a constant sedimentation velocity of around 0.4 mm/h. A platinum electrode was used to avoid the electrolysis phenomenon in this study. Finally, they obtained experiment velocity results that fit the calculation data, and the crystal quality is fair enough. Tran et al. investigated the detailed parameters of electrophoretic CC film formation [78]. They found the voltage to control the sedimentation velocity was important to crystal quality, and the withdrawing rate of the substrate from suspension was also essential to uniformity. Under proper conditions, they obtained over 50 cm^2^ of CC film with good quality in just 5 min.

Not only capillary force can be used in the self-assembly, but also the surface tension during the evaporation of the solvent. The self-assembly induced by evaporation was studied as well. Hong et al. developed an electrospray method to fabricate spherical CCs [79]. The electrospray device could break up those liquid jets subsequently into small droplets by Rayleigh instability. During the evaporation of the solvent, the colloids in droplets self-assembled by capillary force and surface tension, and eventually large CCs spheres (10–40 μm in diameter) were fabricated. Following this idea, Wang et al. proposed a spray-coating method for CCs [80]. When the PMMA latex suspension was sprayed onto the substrate, the surface tension of the solvents would induce the self-assembly of PMMA spheres during the evaporation. In a recent study, Tan et al. combined the direct-write 3D printing technique with evaporation-induced self-assembly, enabling the freeform construction of CCs for the first time [81]. In addition, Li et al. focused on the substrate rather than the colloid suspension [82]. They used the capillary force of the nanoporous substrate to accelerate the assembly of colloids on the surface when printing CCs. This solvent imbibition-induced self-assembly, combined with the meniscus-guided printing technique, made it possible for fast colloidal assembly and patterned printing. Moreover, Díaz-Marín et al. transferred self-assembly colloidal crystals to complex surfaces through capillary peeling [83].

The magnetic-field-force-induced CC self-assembly is also interesting. Ge et al. have discussed the mechanism of magnetic-field-force-induced self-assembly and summarized the application of this method to fabricate CCs from 1D nanochains to 3D films [13]. Despite hard colloids, Wang et al. proved that soft nanospheres like poly(N-isopropyl acrylamide) could also be assembled by magnetic field by adding Fe_3_O_4_ into the non-magnetic nanospheres [84].

### 3.5. Microfluidic Methods

In order to meet the application requirements of micro-CC devices and chips, CCs were reported to be directly assembled in the microfluidic devices in the form of microchannels or microbeads. Yang et al. presented a technique modified from vertical deposition called directed evaporation-induced self-assembly (DEISA) [14]. They fabricated planarized opal-based micro-CC chips successfully, and the morphology can be controlled by a synthetic process. This method first prepared vertically walled, rectangular-shaped microchannels on silicon wafers by deep reactive ion etching. Then these microchannel templates were immersed vertically in monodisperse silica spheres in an alcohol solution to fabricate ordered opal single-crystal structures inside the channels. There is an interesting difference between DEISA and the original vertical deposition in that the strong capillary forces caused by microchannels will force silica spheres to self-assemble only inside the walls rather than at the top of channels. It is obvious that this method shows the potential for the fabrication of optically integrated micro-CC devices for miniaturized applications. And even more optical functionality can be achieved by designing complicated, high-quality opal structures with this method. Zhao et al. prepared colloidal microbeads with the water-in-oil microfluidic device, and the CCs were self-assembled while the solvent droplets evaporated [15,85]. This technique provided a route for the massive production of ordered porous microbeads with uniform sizes for biomedical applications. As for further details on preparation and applications, a comprehensive review has been conducted to summarize the use of CCs in microfluidics [86].

## 4. Ordered Porous Layer Interferometry

Optical interferometry is a technique based on the interference of white light in a thin film (Equation (1)), where the sensing of the interference patterns depends on the optical thickness (OT), the product of film refractive index and physical thickness (OT = nd), so OT changes depend on the joint change of these two parameters of this film in theory [87].

I_λ_ = I_1_ + I_2_ + 2(I_1_I_2_)^1/2^cos2π(2nd/λ) (1)

The I_λ_ is the intensity of reflected light; I_1_ and I_2_ are the intensity of reflected light at the interface; n is the refractive index; d is the film thickness; and λ is the light wavelength. Gauglitz’s group proposed the reflectometric interference spectroscopy (RIFS) technique based on a planar film, which can achieve real-time and in-situ analysis of biomolecular interactions [88,89]. Our group has prepared PS planar interference film by spin-coating and coating it with Ta_2_O_5_ as the substrate for biosensing applications as well [90,91]. For increasing the sensitivity of optical interferometry, Salor’s group reported pSi as the interference substrate prepared by electrochemical etching for DNA biosensing [92]. The porous structure facilitates biomolecular interactions by providing a large surface area, thereby increasing the interaction density of ligands and analytes. Besides, the high refractive index of silicon can provide significant optical contrast between the substrate and solution. However, pSi’s repeatability and mechanical strength restrict its application further [4]. Pan and Rothberg proposed the nanoporous AAO as the interference substrate for biomolecular interaction monitoring [3]. Nevertheless, the reproducibility of the AAO substrate is hard to guarantee. Both pSi and AAO are fabricated by the top-down method of electrochemical etching. As we discussed above, the ordered porous materials can also be prepared through several self-assembly methods, owing to their high reproducibility and low cost. Jiang et al. first reported the Fabry−Pérot fringes in CCs film related to the film thickness, and few studies on this property have been reported since then [9].

**Figure 4:**
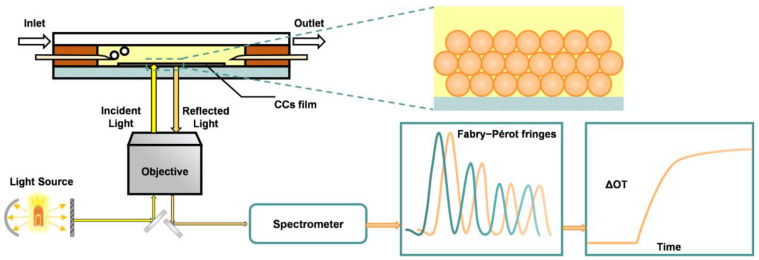
Schematic diagram of the OPLI platform, consisting of a white light source, an inverted microscope, optical fiber, a fiber optical spectrometer, and CCs film.

Our group has optimized the preparation and interference properties of silica CCs films, thoroughly investigating the effects of both colloid size and film thickness, and successfully applied them to biosensing applications [5,93]. In the OPLI platform, the change in the physical thickness of the interferometric substrate due to the biomolecular interactions is negligible compared to the thickness of the interferometric layer. Therefore, the OT variation of the OPLI system depends on the refractive index variation caused by biomolecules, which is also the basis of the application of the OPLI system in general biosensing [94]. Besides the CCs substrate, this OPLI platform combines an inverted microscope with a fiber optic spectrometer for stable and reliable spectra acquisition. (Figure 4) The preparation of self-assembled silica CC films is straightforward, and they possess a highly ordered porous structure that is crucial for reproducibility. The film’s transparency is also a critical property as it allows the acquisition of interference signals from back-reflection, minimizing disturbances caused by turbidity and color in test solutions. Furthermore, the interconnected porous structure facilitates molecular diffusion within the films. These advantages are difficult to achieve when using pSi and AAO as interference substrates. We performed the silica CCs films as scaffolds for biomolecular interactions in various aspects and real-time monitoring in-situ of the interaction with OPLI.

In this section, we will discuss the applications of OPLI in five aspects, including thrombolysis, lipolysis, molecular interactions, complex fluid detection, and other specific scenarios (see Table 1).

### 4.1. Thrombolysis

Monitoring enzymatic hydrolysis can be a challenge for planar interference films, which cannot provide stable support for the substate layer. However, the CCs film can serve as a robust scaffold for facilitating this type of reaction and harbors a plethora of substrate aggregates. Su et al. filled the gelatin into the voids of the silica CCs film and monitored the process of gelatin digestion with trypsin, proving the feasibility of this kind of monitoring [5].

**Figure 5:**
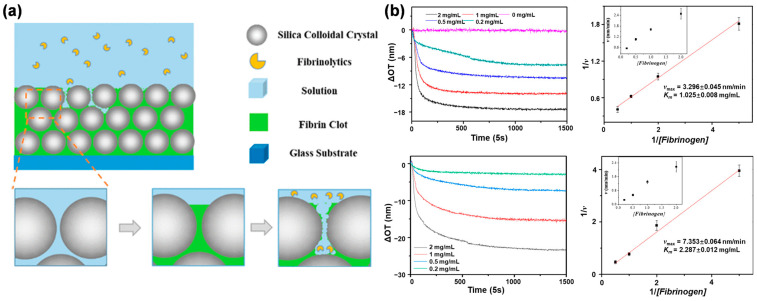
The thrombolysis real-time analysis model for drug tests is constructed on the OPLI platform. (**a**) Diagram of the thrombolysis analysis model. (**b**) The enzyme kinetics analysis. Reproduced with permission from [95]. Copyright (2021) American Chemical Society.

Wu et al. constructed the fibrin layer on silica CCs films as the thrombolysis analysis model for drug tests [95]. (Figure 5) The fibrin gel layer was prepared in a reaction cell on the silica CCs film by polymerization of fibrinogen with thrombin. After the removal of redundant gel on the film surface, the fibrin-loaded silica CCs film still presented good Fabry-Pérot fringes in the spectrum, and the formation of fibrin was proved by the energy dispersive spectrometer (EDS) analysis. The fibrin-loaded silica CCs films were degraded by nattokinase and urokinase, and the whole process was monitored by the OPLI system in real-time. The Michaelis-Menten equation was carried out on the kinetic results of two thrombolysis drugs to obtain the Michaelis constant (K_m_), referred to as their thrombolytic efficacy. Compared to the traditional fibrin plate method, this model provides a valuable tool for real-time investigation of fibrinolysis and further comprehension of its role in both health and disease.

### 4.2. Lipolysis

**Figure 6:**
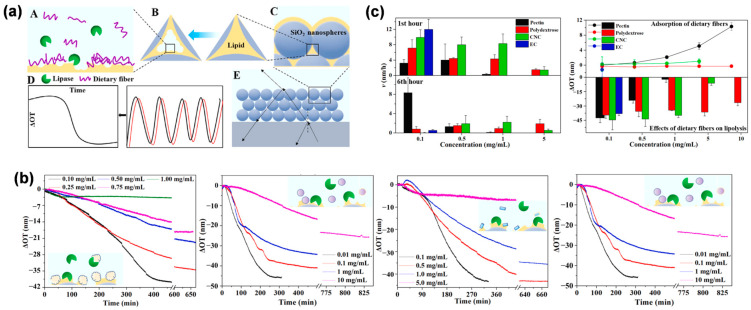
The real-time monitoring of lipolysis through OPLI. (**a**) Diagram of lipid-dietary fiber-lipase interaction on CCs films analyzed by interferometry. (**b**,**c**) Real-time lipolysis process and kinetics influenced by several dietary fibers. Reproduced with permission from [91]. Copyright (2021) Elsevier B.V.

Besides studying thrombolysis on the silica CCs films, the digestion of nutrition can also be investigated through the OPLI platform. Zhou et al. incorporated lipids into silica CC films and investigated the interactions between dietary fibers and lipids, as well as their impact on lipolysis (see Figure 6) [97]. The silica CC films were first surface modified to be hydrophobic with octadecyl trichlorosilane. Then, the glycerol trioleate (GT) was loaded uniformly on the silica CC films by the capillary method, which was proved by the EDS analysis. The baseline of GT-loaded CC films was stable for a long time. The mixture of GT with several kinds of dietary fibers was lipolyzed by lipase and monitored by the OPLI platform to determine the interactions among them. Liu et al. analyzed the different hydrolysis behaviors of vegetable oils that were composed of different nutrients to provide bioavailability information for functional food design [96]. In a similar approach, the release of hydrophobic functional nutraceuticals, such as curcumin, carried by lipids can also be monitored [105], indicating the OPLI platform is capable of conducting research on controlled-release carriers.

### 4.3. Molecular Interactions

The investigation of molecular interactions can be achieved through many kinds of biosensors [106,107,108], and the OPLI can offer a reliable label-free and real-time sensing option for those studies as well.

**Figure 7:**
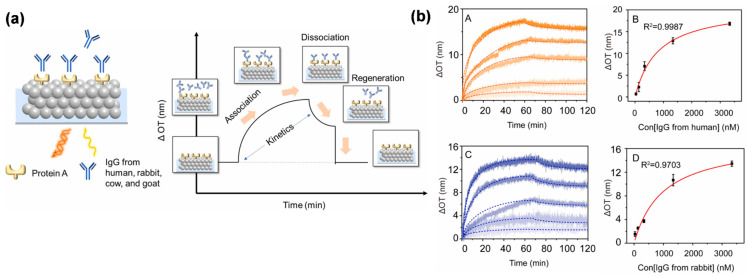
The biomolecular interaction is analyzed in real-time through the OPLI platform. (**a**) Diagram of the interaction between SPA and IgG on silica CCs films. (**b**) The binding kinetics analysis of SPA and IgG from different species. Reproduced with permission from [99]. Copyright (2022) Elsevier B.V.

The storage, transportation, and metabolism of drugs within the human body are crucial aspects of drug development. Understanding the interactions between drugs and proteins is essential for comprehending their toxicity and pharmacodynamic activity in vivo. Wu et al. fabricated the human serum albumin (HSA) immobilized silica CCs films and studied the binding kinetics of HSA with several drugs [6]. The silica CCs films were modified with amino by (3-aminopropyl) triethoxysilane (APTES), followed by glutaraldehyde activation, and then HSA was immobilized on the silica CCs films. Then the OPLI system monitored the binding process of several drugs, including indomethacin, and the equilibrium dissociation constants could be derived from the binding-dissociation curves. Wang et al. studied the interaction between *Staphylococcus aureus* protein A (SPA) and immunoglobulin G (IgG) derived from different species by OPLI as well [99]. This method provides a platform for affinity detection in modern drug development. (Figure 7C) In addition to the drugs, the interaction between polysaccharides and proteins is attracting interest in the fields of biomedicine. The silica CCs films can be modified with polysaccharides, and their interactions with proteins can be studied by OPLI [98,109]. The rapid electrostatic interactions between them were easily monitored and side proved by zeta potential.

### 4.4. Complex Fluid Detection

The real fluidic samples, such as whole blood and milk, are complex with all kinds of disrupting matters, bringing the unpredicted signals into testing, whether the labeled or label-free sensing assays [108]. Pretreating is a common approach for those samples, which is time-consuming and costly. Hence, the direct detection of complex samples in real-time with fewer treatments is essential for post-of-care testing (POCT), medicine development, and agriculture.

**Figure 8:**
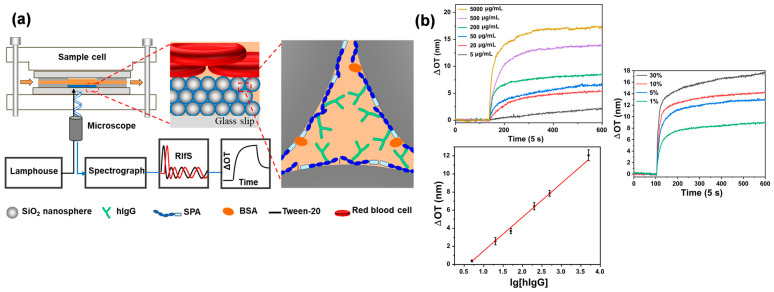
The bioassay detection of whole blood without pretreatment by the OPLI platform. (**a**) The diagram of biomolecular interaction for whole blood IgG content. (**b**) Calibration curve and real-time monitoring results of IgG. Reproduced with permission from [101]. Copyright (2020) American Chemical Society.

The colored fluids were difficult to detect directly on the pSi or AAO sensors. Nevertheless, the transparency property of CC films allows spectrum acquisition from the back, avoiding disturbances from complex samples. Su et al. prepared SPA-functionalized silica CCs films for human immunoglobulin G (hIgG) content detection in whole blood [101]. (Figure 8) The SPA-functionalized silica CCs films were blocked by high-concentration bovine serum albumin (BSA) and 0.02% tween-20 and showed a good performance on anti-nonspecific adsorption. The silica CCs films effectively prevent the entry of large impurities such as blood cells, while the presence of BSA on the surface reduces their adhesion. The hIgG content results obtained from OPLI were highly comparable to those of the immunoturbidimetric assay. This approach can be further applied to the quantification of hIgG concentration in milk and the maintenance of a long storage time of sensors by sucrose protection, which would play an important role in the innovation of novel dairy products [100,102].

In addition to the aforementioned applications, other potential uses can be achieved through the skillful construction of sensitive layers with interferometric optical properties on CCs films. The construction of a hydrogel layer on the silica CCs film can confer its optical interference properties, enabling the study of swelling effects through OPLI and further exploration of controlled release applications for hydrogel drug carriers [104]. Moreover, despite the extensive utilization of OPLI, the silica substrate exhibits insufficient sensitivity towards low doses of biomolecular interactions due to its relatively low refractive index. To address this issue, our research team has also explored the avenue of enhancing surface sensitivity in OPLI systems by utilizing composite liquid crystal sensors to enhance observable refractive index changes of liquid crystals before and after orientation adjustments as optical signals [103].

## 5. Summary and Outlook

We conducted a literature review of recent advances in reflectometric interferometry biosensors based on ordered porous substrates, OPLI. The categories and synthesis of building blocks, the methods of self-assembly, and the applications of CCs in label-free biosensing through interferometry are comprehensively discussed and summarized. First of all, the building blocks play a crucial role in the self-assembly of CCs, and their monodispersity and refractive index significantly impact the quality and optical properties of the resulting crystal. Then, different self-assembly methods operate on distinct principles, each with its own set of advantages and disadvantages. However, they all share the common goal of enhancing crystal quality, enlarging the crystal area, and minimizing preparation time. While the vertical deposition method can offer the best crystal quality for experimental uses, it is difficult to translate this into large-scale industrial production. The most promising method for large-scale production is spin-coating, which can produce CC films with uniform thickness in a short time. However, the quality of CCs obtained by this method is fair and needs to be optimized for various chemical and environmental factors. Meanwhile, the roll-to-roll LB method is also a potential method capable of large-scale production of uniform CCs, and it also has more application scenarios as flexible substrates. Microfluidics-fabricated CCs can serve as natural substrates for miniaturized, high-throughput, multiplex detection instruments in specific biomedical fields. In this review, the primary advantage of OPLI lies in its utilization of self-assembled CCs as an interference substrate. This substrate not only facilitates ease of preparation but also yields highly ordered porous structures with good repeatability. OPLI provides a powerful analytical tool for the study of thrombolysis, lipolysis, drug release, and molecular interactions. The transparent nature of CCs enables the detection light to avoid direct transmission through fluidic samples and instead collect signals from the back of the sensing layer. This greatly reduces interference caused by sample color and turbidity, making it possible for direct detection of complex fluids. Such a capability is highly significant for detecting actual samples.

For the sake of real-world application scenarios, several aspects require further development in OPLI biosensors.

Firstly, specificity. The label-free-based sensors should detect the analytes more selectively. Although the probes immobilized on the substrates can be specific for analytes, the non-specific signals from impurities in less-treated samples can pose challenges for label-free sensors. To the greatest extent possible, surface modification and antifouling measures can minimize non-specific signals.

Secondly, response time. Modern testing requires obtaining results in the shortest possible time, with response time being a crucial metric. The response time of porous substrates, such as CCs, may be slower than that of planar substrates due to mass transfer limitations. However, this problem can potentially be solved by utilizing larger pore sizes. Inversely ordered macroporous substrates can be fabricated using CCs as templates and further applied in OPLI.

Thirdly, miniaturization. The integration of highly sensitive and selective bioassays into compact portable devices can meet the development of the POCT industry. Portable fiber optical spectrometers are suitable for such signal acquisition. Microfluidics can provide a promising approach for biosensing platform miniaturization by constructing the sensing layer in microchannels. Besides, multiplexing and high-throughput testing can also be achieved through microfluidics. Still, the balance of sensitivity, time consumption, stability, and reliability should be a concern when developing such portable test devices. 

Fourthly, complex body fluid samples. The POCT can be mainstreamed for future medical care outside hospital laboratories. Although the major challenge for such equipment is reliability for analyzing complex body fluid samples. There are many biomarkers in a single sample that contains many kinds of impurities. Refining the surface modification or incorporating sample pretreatment through lab-on-a-chip techniques can potentially advance this field by preventing non-specific adsorption.

Finally, simulation of biomolecular interactions in the body’s environment. With the further complexity of biological science in recent years, especially in the post-pandemic era, the study of the mechanisms of biomolecular interaction may face new challenges. The interaction of biomolecules in a real biological environment may be affected by multiple factors, which in turn can lead to deviations from in vitro experiments. Therefore, establishing a simulated body environment on biosensors may be a solution to this problem. For example, the human respiratory mucosa system is a complex porous structure with different types of mucins that can prevent pathogen invasion [110]. The OPLI technique may provide a possible study path through adjusting the pore size of the CCs films and surface modification to study the biomolecular interactions in the mucosa.

## Figures and Tables

**Table 1:** Overview of typical biosensing applications through OPLI.

Scenarios	Surface Modification	Analytes	Limit of Detection	Mechanism	Refs.
Enzymatic hydrolysis	Fibrin-loaded	Nattokinase	0.75 U/mL	Mass decreasing	[95]
	Lipid-loaded	Lipase	N/A	Mass decreasing	[96,97]
	Chitosan-loaded	Pepsin	N/A	Mass decreasing	[98]
Molecular Interactions	human serum albumin (HSA)-immobilized	Indomethacin, warfarin	N/A	Mass increasing	[6]
	*Staphylococcus aureus* protein A (SPA)-immobilized	Human immunoglobin G (hIgG)	1 μg/mL	Mass increasing	[99,100]
Complex fluids	SPA-immobilized	hIgG (in whole blood)	5 μg/mL	Mass increasing	[101]
		hIgG (in milk)	1 μg/mL		[102]
Molecular orientation	Liquid crystal-loaded	DTAB (surfactant)	50 μmol/L	Liquid crystal orientation	[103]
Controlled release	Chitosan hydrogel-embedded	Indomethacin	N/A	Hydrogel-swelling	[104]

## Data Availability

No new data were created or analyzed in this study. Data sharing is not applicable to this article.
